# Cortical inflammation and brain signs of high-risk atherosclerosis in a non-human primate model

**DOI:** 10.1093/braincomms/fcab064

**Published:** 2021-04-01

**Authors:** Vanessa Di Cataldo, Justine Debatisse, Joao Piraquive, Alain Géloën, Clément Grandin, Michaël Verset, Fabrice Taborik, Emmanuel Labaronne, Emmanuelle Loizon, Antoine Millon, Pauline Mury, Vincent Pialoux, André Serusclat, Franck Lamberton, Danielle Ibarrola, Franck Lavenne, Didier Le Bars, Thomas Troalen, Joachim Confais, Claire Crola Da Silva, Laura Mechtouff, Hugues Contamin, Zahi A Fayad, Emmanuelle Canet-Soulas

**Affiliations:** 1 CarMeN Laboratory, Univ Lyon, INSERM U1060, INRAE 1397, Université Claude Bernard Lyon 1, Lyon, France; 2 Siemens-Healthcare SAS, Saint-Denis, France; 3 CERMEP—Imagerie du Vivant, Lyon, France; 4 Cynbiose SAS, Marcy-L'Etoile, France; 5 LIBM Laboratory, Univ Lyon, Université Lyon 1, Lyon, France; 6 Radiology Department, Louis Pradel Hospital, Hospices Civils de Lyon, Lyon, France; 7 Stroke Department, Hospices Civils de Lyon, Lyon, France; 8 BioMedical Engineering and Imaging Institute, Icahn School of Medicine at Mount Sinai, New York, NY, USA

**Keywords:** atherosclerosis, aging, neuroinflammation, stroke, choroid plexus

## Abstract

Atherosclerosis is a chronic systemic inflammatory disease, inducing cardiovascular and cerebrovascular acute events. A role of neuroinflammation is suspected, but not yet investigated in the gyrencephalic brain and the related activity at blood−brain interfaces is unknown. A non-human primate model of advanced atherosclerosis was first established using longitudinal blood samples, multimodal imaging and gene analysis in aged animals. Non-human primate carotid lesions were compared with human carotid endarterectomy samples. During the whole-body imaging session, imaging of neuroinflammation and choroid plexus function was performed. Advanced plaques were present in multiple sites, premature deaths occurred and downstream lesions (myocardial fibrosis, lacunar stroke) were present in this model. Vascular lesions were similar to in humans: high plaque activity on PET and MRI imaging and systemic inflammation (high plasma C-reactive protein levels: 42 ± 14 µg/ml). We also found the same gene association (metabolic, inflammatory and anti-inflammatory markers) as in patients with similar histological features. Metabolic imaging localized abnormal brain glucose metabolism in the frontal cortex. It corresponded to cortical neuro-inflammation (PET imaging) that correlated with C-reactive protein level. Multimodal imaging also revealed pronounced choroid plexus function impairment in aging atherosclerotic non-human primates. In conclusion, multimodal whole-body inflammation exploration at the vascular level and blood−brain interfaces identified high-risk aging atherosclerosis. These results open the way for systemic and central inflammation targeting in atherosclerosis in the new era of immunotherapy.

## Teaser

Elevated cortical inflammation and defective choroid plexus activity associate with high-risk atherosclerosis in cynomolgus monkeys. Using diet-induced atherosclerosis, in-vivo imaging and gene analysis, Di Cataldo et al. showed (i) monkeys exhibit similar atherosclerosis features and vulnerable plaques-associated genes as patients, (ii) brain imaging demonstrated frontal cortex neuroinflammation and neurovascular signs.

## Graphical Abstract

**Graphical Abstract:**
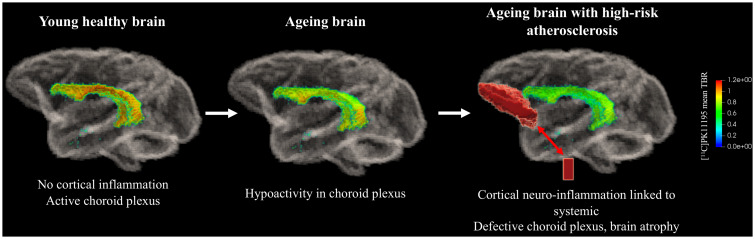

## Introduction

Atherosclerosis is an inflammatory disease^1^ that is just entering the immunomodulation therapy era for personalized care.^2^ Whereas the contribution of systemic and cardiometabolic inflammation is well recognized, localized neuroinflammation has only recently been incriminated.^3^

There is a translational gap between mouse models and men, especially in atherosclerosis.^4^ Firstly, plaque rupture and cardiovascular events or stroke are not frequent in mouse models.^5^ Moreover, the gyrencephalic brain organization and higher white-to-grey matter ratio in humans are thought to play a major role in neuroinflammation processes.^6^^,^^7^ The cynomolgus macaque under atherogenic diet has been found to be invaluable in establishing the protective effect of oestrogen against coronary plaque development.^8^ In this model, atherosclerosis develops with a lipidic blood profile and multi-site progression as in humans, and the carotid lesions reproduce the plaque morphology observed in clinical studies, as vessel geometry is very close to that found in humans.^9^ Moreover, the innate immune system is a key player in vulnerable plaque, and this non-human primate (NHP) atherosclerosis model is the only one to display the same chemokine and cytokine armamentarium as in human atherosclerosis.

Recent epidemiological studies showed the relationship between atherosclerosis, systemic inflammation and cognitive dysfunction, but did not specifically address neuroinflammation issues.^10^ To our knowledge, only one small clinical study reported cerebrovascular inflammation imaging in three patients after acute myocardial infarction.^3^ Neuroinflammation has never been specifically explored in an atherosclerosis model with gyrencephalic brain, a key issue for human translation in neurovascular diseases.^11^

The objective of the present study was to explore advanced atherosclerosis in aged NHPs using a unique combination of multimodal multisite imaging and gene analysis. We hypothesized that neuro-inflammation and modulation of choroid plexus (CP) activity are strongly involved in high-risk atherosclerosis.

## Materials and methods

### NHP model of atherosclerosis

All animal studies and experiments were approved by the local and national authorities and carried out in accordance with European Directive 2010/63/UE and the ARRIVE (Animal Research: Reporting in Vivo Experiments) guidelines, after approval by the local institutional review board (#1367 and #1239). Every effort was made to minimize animal suffering and reduce the number of animals used in the experiments. Animals were acclimatized for at least 10 days prior to the first day of study and were housed collectively with the following ambient parameters: aeration with >10 air changes/h and no air recirculation, 12-h light/12-h dark photoperiod, room temperature 22 ± 3°C and humidity 55 ± 20%. The animal room and cages were cleaned daily. In this observational study, 13 aged cynomolgus monkeys from Philippines or Mauritius (*Macaca fascicularis*, 3 males, 10 ovariectomized females) were included in the diet intervention. They received high-fat high-cholesterol diet [HC: 23% fat (w/w), 0.5% cholesterol (w/w), 11.3% saturated fatty acids (w/w); E39126-34, Ssniff, Germany] (*n* = 13) for 24 months. The control group of aged animals comprised three animals (*Macaca fascicularis*, two ovariectomized females and one male) under standard diet [SD: 11% fat (w/w), saturated fatty acids <1%; Pri V3944-000, Ssniff, Germany] for 24 months. Food rations were adapted to each individual according to body weight (100 g/day/animal under 5 kg; 200 g/day/animal over 5 kg). One fruit was provided daily for each animal. Treats were also occasionally given at the end of the day as part of the test facility's environmental enrichment program. In the HC group, six serial blood samples were collected, and two ultrasound (US) exams of the carotids were made to evaluate lipid profiles and atherosclerosis development. Primary outcome was the presence of at least one atherosclerotic lesion in carotids or aorta detected by US imaging. At end of study, the animals were deeply anesthetized before lethal injection of pentobarbital. Carotids, aortic arch, abdominal aorta and non-vascular tissues (heart, pericardial and pericoronary, visceral and subcutaneous fat, brain) were collected and prepared for further analyses (i.e. pathological examination, gene expression or biochemistry measurement). Investigators were blind to the SD or HD assignment during imaging experiments and data analysis. Exclusion criteria during the study were premature death. Animals without follow-up or with insufficient imaging data quality were removed from the analysis.

Four young control (YC) animals (YC, *Macaca fascicularis*, Mauritius; mean age, 7 ± 0 years; males) under SD served as controls for inflammation. They underwent a blood sample for hsCRP measurement and a brain and neck PET [^11^C]PK11195 exam with MRI pre- and post-gadolinium injection.

### Longitudinal follow-up protocol

**Figure 1:**
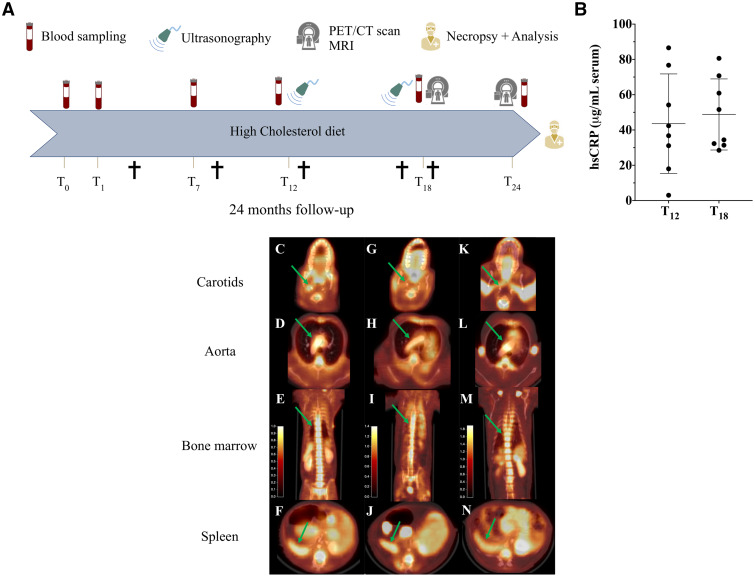
**Longitudinal characterization of an NHP model of atherosclerosis with imaging and blood biomarkers.** Experimental study design (**A**) over the 24 months (†, five premature deaths during the protocol). High level of systemic inflammation was present in old HC NHPs during follow-up (hsCRP >2 µg/ml at 12 and 18 months; T+12 and T+18, respectively, *n* = 8 NHPs) (**B**) Higher FDG uptake (arrows) in carotids, aortic arch, bone marrow and spleen in an at-risk HC NHP (HC #9, hsCRP =71 µg/ml) (**C−F**), compared with a ‘lower risk’ HC NHP (HC#5, hsCRP = 31 µg/ml) (**G−J**), and an old SD NHP (SD#1, hsCRP=18 µg/ml) (**K−N**). HC = high cholesterol group; hsCRP = high-sensitivity C reactive protein; NHP = non-human primate; SD = standard diet group.

For SD animals, one blood sample was taken at end of study and, for HC animals, six during follow-up (Fig. 1A). Animals were fasted and samples were taken from the femoral vein under anaesthesia by ketamine (Imalgene^®^ 1000, Merial, France). Blood was collected in EDTA tubes for plasma harvesting and in citrate tubes for cholesterol/lipoprotein assay. The EDTA and citrate tubes were centrifuged at 1500 g for 10 min; then supernatant was removed and stored at −80°C until further assays. Total plasma cholesterol and triglycerides were measured using the Accutrend Plus kit (Roche, France), and lipoprotein fractions in plasma were assessed using the Lipoprint^®^ LDL subfractions kit (Quantimetrix, CA, USA) according to the manufacturer’s instructions. At the last time points (12−24 months), high-sensitivity C-reactive protein (hsCRP) was assessed in serum using a commercial kit (CSB-E10035Mo, CUSABIO, Baltimore, MD, USA).

US was performed at 12 and 18 months (Fig. 1A) by an experienced cardiologist and veterinarian. A Philips CX 50 apparatus with C8-5 microconvex probe was used according to a standardized imaging protocol: aorta, iliac bifurcation and carotid arteries. Results were expressed as presence or absence of lesion, and vessel wall thickness and intima-media thickness when possible (otherwise, in patients, intima-media thickness >0.7 mm can be assumed). For longitudinal assessment of disease progression, a score was given at each US session: 1 for normal, 2 for small lesion in a single location, 3 for multi-site small lesions, and 4 for multi-site lesions with at least 1 large plaque.

### Pathology

For histopathology, the left carotid and parts of the aortic arch and heart (apical area) were removed and stored in 4% paraformaldehyde solution for 24 h, then in 15% sucrose for 48 h, and placed in a histology cassette and frozen in liquid nitrogen, except for heart samples, which were embedded in paraffin. The samples were cut in 10 µm slices by cryostat (LEICA CM3050S), and then stained with haematoxylin/eosin (MHS32-1L and HT110232-1L, Sigma-Aldrich), Oil Red O (841K04010169, Merk) and Masson trichrome (ab150686, AbCam) to visualize, respectively, section morphology, lipids and fibrosis. Photographs were acquired using the Zeiss Scope A1 microscope (Zeiss). A researcher experienced in vascular pathology and blind to the imaging results examined all histology sections of each specimen. The following features were graded on a simple semi-quantitative scale, previously published by Lovett et al.^12^: thrombus area; thick, thin (<200 μm) or ruptured fibrous cap (FC); intra-plaque haemorrhage (IPH); neovascularization; and macrophage infiltration. Loose fibrosis, defined as fibrous tissue rich in non-fibrillar extracellular matrix with thin non-condensed collagen fibres, was graded as <30% or >30% of total fibrous tissue. Plaques were classified according to the American Heart Association classification of coronary atherosclerosis and according to the Lovett and Redgrave classification.^12^^,^^13^

For translation, the same histology and gene analysis was performed in NHP and in human carotid endarterectomy from symptomatic and asymptomatic patients. Human carotid samples (*n* = 19), taken from a larger clinical study,^14^ were obtained from asymptomatic and symptomatic patients undergoing carotid endarterectomy in the vascular surgery department of Edouard Herriot Hospital (Lyon, France). Written informed consent for analysis of blood and tissue samples was obtained from all patients before surgery. Patients were considered symptomatic if an ipsilateral carotid-related neurological event was reported in the previous 6 months. Samples were collected and prepared for gene expression and pathological analysis. Patient data are summarized in Supplementary Table 1. Endarterectomy samples were prepared using standard methods in our institution^15^ and analysed by an experienced pathologist.

### mRNA analysis

mRNA was extracted from snap-frozen vascular and tissue samples using the TRIZOL reagent procedure. Gene expression of consensual markers of metabolism (including *tspo* and *Hk1* related to imaging tracers) and macrophages was assessed in parallel on NHP carotids and aorta and on endarterectomy samples from patients with symptomatic or asymptomatic carotid stenosis. Expression was assessed for 20 genes corresponding to glycolytic metabolism (*Hk1*), mitochondria metabolism (*Ppif, Tspo*), pan-macrophages (*Cd14, Cd68*), M1 (*Il-1β, Tlr4, Ccl2, Il-6, Tnfα, Cxcl9, Il-17ra, IL-22ra*) and M2 (*Il-1ra, Ccr2, Il-10, Clec7a, Irf4, Cd163*) markers and lymphocyte infiltration (*Cd3ε*) (list of primers: Supplementary Table 2), using the qPCR method. TBP, ARL1 and Actin β were evaluated and Actin β was validated as the reference gene in the various tissues after careful evaluation of sample quality and mRNA content. Heat-maps for genomic risk profile were generated by hierarchical clustering of samples, using the Ward2 algorithm (R software).

### Dual tracer PET and MRI imaging for carotid and brain

[^18^F]FDG and [^11^C]PK11195 PET images were acquired on a 64-multidetector Biograph mCT PET/CT scanner (Siemens Healthcare SAS, Erlangen, Germany) with an axial field of view of 22 cm. The scanner was checked every day using established calibration procedures.

#### Whole body [^18^F]FDG imaging

Prior to [^18^F]FDG PET, fasting blood glucose concentration was recorded to check for a level <200 mg/dl. [^18^F]FDG imaging was performed in all animals 60 min after intravenous [^18^F]FDG injection of a target dose of 5 MBq/kg (mean dose, 86.24 ± 22.72 MBq). Animals were placed in supine position. Sixty minutes after injection of the radiotracer, a whole-body low-dose CT scan (80 kV, 20 mAs, 1 mm slice thickness and 0.5 s pitch) was acquired for attenuation correction. The CT scan dose was adjusted using Care Dose 4D software (Siemens Healthcare SAS), with the animal's body centred in the scanner to enable whole-body coverage. Three bed positions were used and acquired for 5 min each: 1 centred on the brain and the carotids, encompassing the aortic arch and its branches, and the other 2 distally to cover the upper and lower part of the abdomen. All emission images were normalized using an inhomogeneity detector and corrected for dead time, random coincidences, diffusion and attenuation. Image reconstruction was performed with iterative ordered-subset expectation maximization (iterative OSEM method, TrueX + TOF UltraHD-PET) with 12 iterations and 21 subsets (number of iterations, ENI of 252), non-filtered in line with recent recommendations for FDG analysis in atherosclerosis.^16^ FDG PET data were reconstructed with a voxel size of 4.07 × 4.07 × 2.03 mm^3^.

After the PET examination, CT angiography was acquired with the same field of view (FoV). A bolus of 20 ml Iomeron 400 (Guerbet, Aulnay-sous-Bois, France) was injected at a rate of 3.5 ml/s in the antecubital vein, followed by saline flush at the same rate. Acquisition parameters were 80 kV, 20m As, FOV 500 mm, 30 s B filter, slice thickness 1 mm, and pitch 0.5 s.

#### [^11^C]PK11195 imaging

A subgroup of five animals (HC: N = 3, SD: N = 2) were also explored using the [^11^C]PK11195 PET tracer (mean dose, 123.70 ± 30.96 MBq) targeting TSPO of activated macrophages. Thanks to its shorter radioactive half-life, this [^11^C]PK11195 examination was performed before the FDG examination. PET emission data of [^11^C]PK11195 centred on the carotids were acquired over 60 min in list-mode format and rebinned into 18 temporal frames: 11.16 s, 6 × 10 s, 4 × 60 s, 6 × 300 s and 2 × 600 s. An additional [^11^C]PK11195 dataset of four YC animals acquired over 60 min in list-mode format on a Biograph mMR scanner (Siemens Healthcare SAS) was used (mean dose, 135.5 ± 28.10 MBq). YC [^11^C]PK11195 PET data were reconstructed on a 256 × 256 matrix (voxel size: 0.7 × 0.7 × 2.03mm^3^), 30 cm FoV using a point-spread function iterative reconstruction method including correction for scatter, random counts and dead time. Dynamic scans were corrected for attenuation using CT-based attenuation map and were reconstructed in 25 frames: 6 × 10 s, 6 × 20 s, 6 × 120 s and 8 × 300 s.

#### Magnetic resonance imaging

MRI was performed on a MR scanner (MAGNETOM Prisma, Siemens Healthcare SAS). Animals were installed in supine position, with the head in the centre of the posterior part of the 64-channel head-neck coil.

For carotid imaging, a four-channel phased array receiver coil was combined with the head coil to optimize signal-to-noise ratio and with cardiac triggering and breathing monitoring. The carotid was located with time-of-flight (ToF) sequences. High resolution MRI images were centred on the bifurcation and acquired using turbo-spin echo (TSE). Proton density weighted (PDW) and T_1_-weighted (T1W) images were used and acquisition parameters are detailed in Supplementary Table 3. Dynamic images were acquired with the TWIST (Time-resolved angiography With Interleaved Stochastic Trajectories) pulse sequences and using all MR coil channels (Supplementary Table 3). TWIST sequences were applied in the coronal orientation and acquired with a separation of 5 s between frames, interpolated to 2.46 ms. A bolus of gadolinium contrast agent (0.1 mmol Gd/kg, DOTAREM^®^, Guerbet, Aulnay-sous-Bois) was administered intravenously.

Brain imaging was performed with the 40-channel phased-array head-neck coil (posterior part of the coil) and the following sequences were acquired: 2D FLAIR (Fluid Attenuation Inversion Recovery), 2D T2* gradient echo, 3D T1 MPR (Multiplanar Reconstruction). Acquisition parameters are detailed in Supplementary Table 3.

### Vascular image analysis

For MRI, vessel wall area was measured (in mm^2^) in the right and left carotids (three slices per artery) by manual delineation of the inner and outer contours. MR angiography and CT angiography acquisitions then registered the region of interest (ROI) on the PET/CT data (Supplementary Fig. 1).

For whole-body [^18^F]FDG, standardized uptake value (SUV) images were computed for each [^18^F]FDG scan. Target-to-background ratio (TBR:SUV_max_ normalized by SUV_max_ of the superior vena cava) was measured at three vascular locations (right and left carotids, aortic arch and abdominal aorta at the renal bifurcations).

### Brain image analysis

For brain [^18^F]FDG image post-processing, SUV images were computed for each [^18^F]FDG scan and PET images were co-registered to the 3D T1 MPR. T_1_-weighted (T1w) imaging data were also used to calculate a transformation matrix, using MincTools and in-house optimization tools (https://bic-mni.github.io/). Non-linear registration was used to co-register imaging data on a common space using a *Macaca fascicularis* 3D template.^17^ A 79-label atlas covering all the brain was used to anatomically sample brain [^18^F]FDG SUV_mean_ and SUV_max_ in the labels. Some atlas labels were merged into larger brain structures (frontal lobe, basal ganglia, limbic structures etc.) following brain segmentation as defined previously.^17^

For brain [^11^C]PK11195 analysis, levels of translocator protein (18 kDa) expressed in active microglia/macrophages were assessed using [^11^C]PK11195 analysis. The last 30 min of each scan (i.e. 30−60 min post-injection) were averaged and used for analysis. SUVs were computed and TBR (Tissue to Background Ratio) was obtained by dividing SUV images by mean cerebellar SUV. As described earlier, PET images were co-registered to each animal’s 3D T1 MRI and to the common template space. [^11^C]PK11195 SUV_mean_, SUV_max_ and TBR_mean_ and TBR_max_ were computed by anatomically sampling SUV and TBR images using the 79-label atlas covering all the brain.

To further investigate TSPO expression across animal groups, parametric mapping analysis was performed using SPM12. Each HC and SD [^11^C]PK11195 TBR image was individually compared with the dataset of four YC [^11^C]PK11195 TBR images using a 2-sample *t*-test. *T*-score maps were computed and divided between ‘[^11^C]PK11195 hypofixation’ for negative *T*-score and ‘[^11^C]PK11195 hyperfixation’ for positive *T*-score. The *T*-score maps were also anatomically sampled using the previously described 79-label atlas for heat-map representation.

### Statistical analysis

Values are expressed as mean ± standard deviation or median with percentile ranges. Bivariate comparisons were made using the Student *t* test for continuous variables or Wilcoxon test as appropriate. Spearman’s rank test was used to assess correlation between gene expression levels. A *P*-level < 0.05 was considered significant. For comparison between three or more independent groups, one-way analysis of variance (ANOVA) with *posthoc* Bonferroni correction was performed, or Kruskal−Wallis ANOVA with Dunn’s multiple comparisons when distribution was not normal. Statistical analyses were performed using GraphPad Prism v9.0 (GraphPad Software, La Jolla, CA, USA).

### Data availability

All data generated or analysed during this study are included in this published article and its Supplementary Materials.

## Results

### NHP model of high-risk atherosclerosis validated by imaging and blood markers

There were sixteen old cynomolgus monkeys (13 HC and 3 SD) of 13.1 ± 4 years at the beginning of the study (5−7 years for the males and 12−18 years for the females). The lipid profile changed rapidly after initiating the HC diet, with elevated levels of both triglyceride and total cholesterol until the end of the study (Table 1; Supplementary Fig. 2). Both HDL cholesterol and the HDL/LDL ratio decreased significantly. Sub-fraction lipoprotein analysis confirmed an at-risk lipoprotein profile. With carotid US, most HC animals displayed lesions similar to humans, with progression from 12 to 18 months (Supplementary Fig. 2). The most predictive inflammatory marker in clinical studies is elevated C-reactive protein, measured on high-sensitivity assay (clinical threshold, hsCRP> 2 µg/ml).^2^ In contrast to atherosclerosis mouse models,^18^ hsCRP is a highly relevant inflammation biomarker in NHP models.^19^ All HC animals had persistent hsCRP elevation from 12 to 24 months (42 ± 14 µg/ml at 24 months) (Fig. 1B; Supplementary Fig. 2).

**Table 1:** Description of HC and SD old NHPs at end of study (following American Heart Association recommendations, males and females were included^4^)

	HC (*n* = 8)	SD (*n* = 3)
Male/female	2/6	1/2
Age (years)	14 ± 3.5	15.7 ± 5.9
BW (kg)	5.7 ± 1.4	6.3 ± 2.2
BW gain (kg)	1.3 ± 0.8	N/A
High TG (>200 mg/dL)	4/8	1/3
High Chol (>240 mg/dL)	8/8	0/3
Low HDL/LDL (<0.3)	7/8	0/3
hsCRP (µg/mL)	42.3 ± 13.9	29.2 ± 9.4
Progressive carotid lesion (US)	3/8	N/A
Pathology findings		
Advanced carotid plaque	5/7	0/3
Large lipid core (>50%)	4/7	0/3
Thin or ruptured FC	1/7	0/3
Coronary/aorta plaques	5/8	1/3

BW = body weight; Chol = total cholesterol; HC = high cholesterol group; hsCRP = high-sensitivity C reactive protein; NHP = non-human primate; SD = standard diet group; TG = triglyceride; US = ultrasound imaging; N/A = not applicable.

During the 24 months of the study, 5 HC animals died (Fig. 1A): 2 early and abruptly after diet initiation, 2 euthanized because of acute kidney failure and 1 prematurely from acute pancreatitis (severe pancreas autolysis found on necropsy). In these animals, the hsCRP level was high (Supplementary Fig. 2) and large atherosclerotic lesions were also observed in various vascular beds on necropsy.

We performed whole-body [^18^F]FDG positron emission tomography (PET) after 18 months’ diet (Fig. 1A) to evaluate inflammation in different vascular beds. We also analysed in haematopoietic organs (bone marrow, spleen) as it was recently demonstrated as an important feature of the high-risk human pathology.^20^ HC animals had high [^18^F]FDG activity in the various atherosclerotic locations (carotids and aortic arch), and in bone marrow and spleen (Fig. 1C−J).

Thus, an animal model of high cardiovascular risk was obtained, with fatal events, elevated hsCRP, advanced lesions and systemic inflammation in multiple vascular locations and in haematopoietic tissues.

### Histology and gene analysis confirmed high-risk metabolic and inflammatory imaging

**Figure 2:**
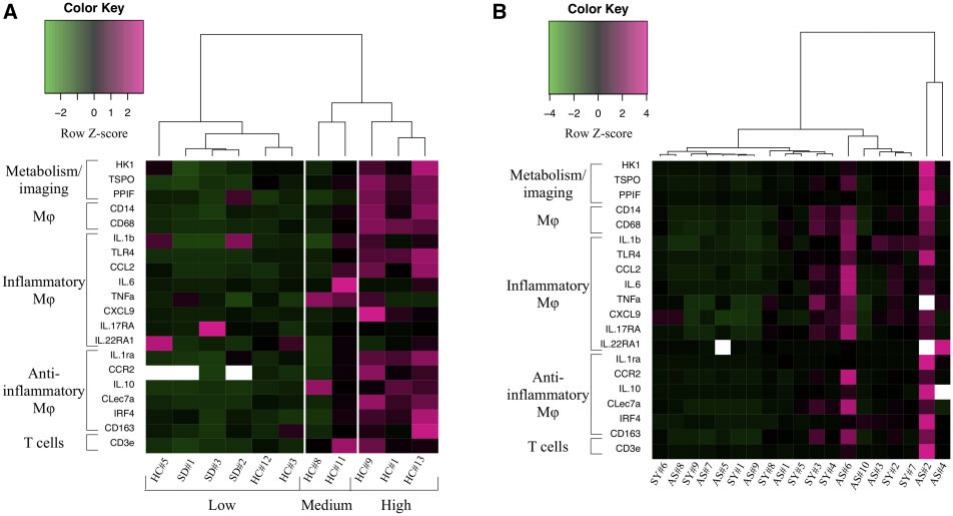
**Carotid gene expression similarities with human samples identify high-risk profiles.** mRNA expression levels of selected vulnerable plaque markers in the carotid arteries divided HC (*n* = 8) and SD (*n* = 3) NHPs into three groups according to expression levels (**A**). mRNA expression levels of the same markers in the patients’ endarterectomy samples confirmed the similarity of patterns with significant co-expression of ‘imaging-related’ genes (Hk1, TSPO), inflammatory and anti-inflammatory genes in asymptomatic (*n* = 10) and symptomatic patients (*n* = 9) (**B**) (heat map row *z*-score of normalized gene expression and Ward2 hierarchical clustering).

**Figure 3:**
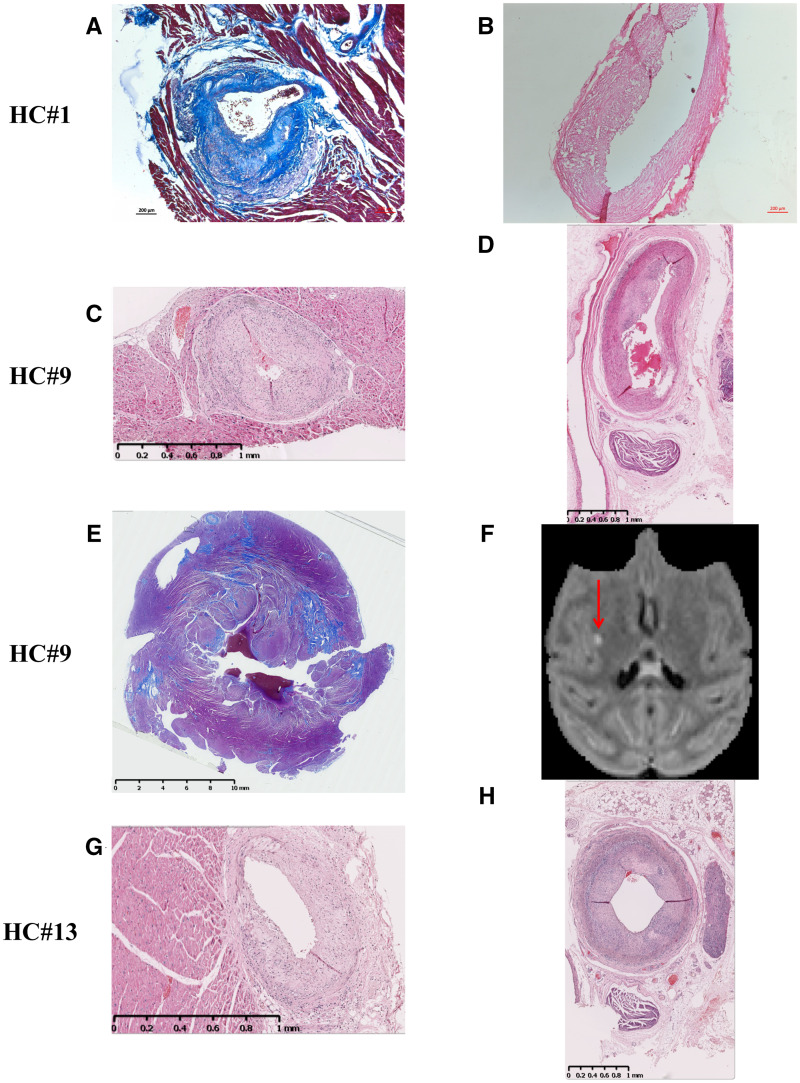
**Pathological findings in NHP confirmed high-risk profiles.** The three NHPs (HC#1, HC#9, HC#13) identified from gene analysis presented high-risk features with advanced plaques either in coronary or carotids and downstream events (**A**−**H**). Histological evidence of coronary stenosis (**A, C, G**), and severe and/or diffuse left carotid plaques (**B, D, H**), with myocardial fibrosis on histology (**E**) and brain MRI showing a lacunar stroke (**F**). NHP = non-human primate; HC = high cholesterol group; SD = standard diet group.

Histology and gene analysis were performed to categorize active carotid lesions. For translation, human carotid samples (*n* = 19) from a larger clinical study^14^ were obtained from asymptomatic and symptomatic patients undergoing carotid endarterectomy in the vascular surgery department (Supplementary Table 1 for patients’ characteristics). For mRNA analysis, we selected consensual pro- (M1) and anti-inflammatory (M2) genes according to recent findings in human atherosclerosis^21^^,^^22^ and genes related to imaging markers and metabolism: i.e. HK1 for [^18^F]FDG and TSPO for [^11^C]PK11195 (Fig. 2A and B). In both NHPs and patients, plaque histology showed similar features of vulnerable plaques (Supplementary Fig. 3) with complex composition, lipid core and inflammatory cells. Intra-plaque gene cluster analysis showed similar inflammation patterns in NHPs and patients (Fig. 2A and B). In both, there were strong relations between expression of ‘imaging-related’ genes (Hk1 for [^18^F]FDG and TSPO for [^11^C]PK11195) and vulnerable plaque ones such as Ccl2, Il-1β and Il-6 (Fig. 2A and B; Supplementary Tables 4 and 5), but also with anti-inflammatory genes. These results confirmed the translational value of the pattern, observed in NHPs, of high/low profiles corresponding to histological features of active/more stable lesions (Supplementary Fig. 3). High co-expression was found in 27% of NHPs and 44% of symptomatic and 20% of asymptomatic patients, corresponding in both NHPs and asymptomatic patients to at-risk histological features. The NHPs grouped as high by mRNA analysis (3 out of 11 cases) (Fig. 2A) presented high-risk features with advanced vascular lesions and downstream events (myocardial fibrosis, lacunar stroke) (HC #1, 9 and 13) (Fig. 3A−H). Vulnerable features such as eccentric plaques with shoulder and thin FC were present in the coronaries of these HC NHPs (Figs 2D−H and 3B). Progression was confirmed on US and circulating biomarkers (LDL-C and hsCRP) (Fig. 1; Supplementary Fig. 2). Of note, one of the aged NHPs under standard diet had high CRP, vascular inflammation and atherosclerosis in one location, heart inflammation and myocardial fibrosis (Table 1; Supplementary Figs 3 and 4). The gene association between metabolic, inflammatory and anti-inflammatory genes was confirmed in the other two atherosclerosis locations (aortic arch and abdominal aorta) (Supplementary Fig. 3). In downstream organs (frontal cortex and myocardial apex), heatmap gene expression confirmed the association and high expression in two to three subjects (Supplementary Fig. 4). Finally, mRNA expression in NHP visceral and pericardial adipose tissue showed associated inflammation similar to metabolic syndrome (Supplementary Fig. 4). The positive relationship between hsCRP and triglyceride level at end of study (*r* = 0.8, *P* < 0.05) and [^18^F]FDG imaging confirmed the inflammatory status of the visceral compared with the subcutaneous fat (Supplementary Fig. 4). These findings confirmed the metabolic syndrome with active systemic inflammation in this atherosclerosis model.

### Active atherosclerosis results in neuroinflammation signatures, brain atrophy, and CP silencing

**Figure 4:**
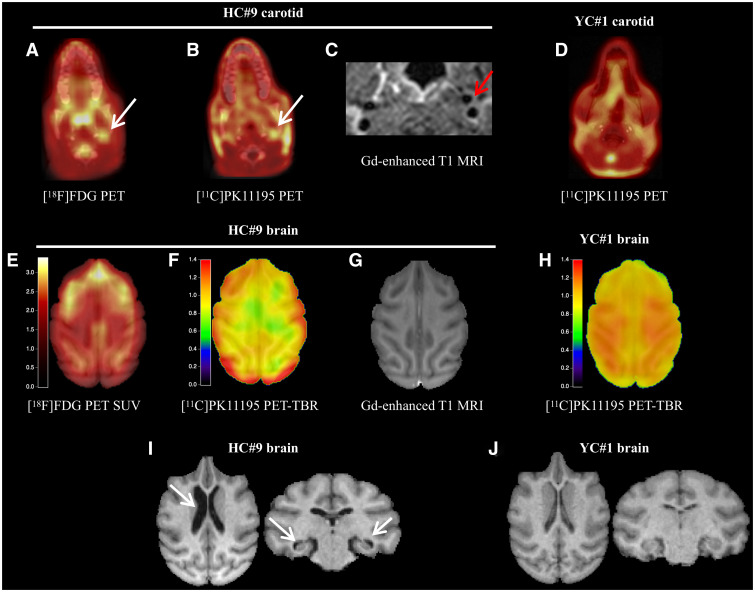
**PET and MRI demonstrate localized inflammation in carotid and brain, and brain atrophy in atherosclerotic NHPs.** Dual tracer PET in carotids and brain showing colocalized FDG (**A**) and PK11195 uptake (**B**) in carotid plaque together with gadolinium MRI enhancement (**C**) in HC#9 and no uptake in YC animal (YC#1—**D**). Brain uptake shows high localized FDG uptake in the frontal lobe and PK11195 uptake was heterogenous across brain (**E** and **F**) with no gadolinium MRI enhancement (**G**). For comparison, brain imaging in a YC animal (YC#1) shows no localized PK11195 uptake (**H**). Ventricle enlargement and hippocampal atrophy were found in HC#9 (**I**, arrows) compared with YC#1 (**J**). HC = high cholesterol group; NHP = non-human primate; YC = young control group.

In search of neuroinflammation associated to active atherosclerosis, we performed combined vascular and brain inflammation imaging. A subgroup of five animals were studied with dual [^18^F]FDG/[^11^C]PK11195 tracer imaging. Carotid and brain MRI was also acquired at end of study to measure final carotid plaque area and to evaluate plaque permeability and blood-brain barrier (BBB) damage on gadolinium-enhanced T1 imaging. We used a control dataset of 4 young animals for hsCRP assay, brain [^11^C]PK11195 PET and gadolinium-enhanced MRI, to obtain systemic and cerebral reference levels. In HC animals, MRI and PET/CT vessel wall analysis (Supplementary Fig. 1) confirmed advanced plaques and carotid plaque activity on both [^18^F]FDG and [^11^C]PK11195 and gadolinium-enhanced MRI (Fig. 4A−C). In YCs, hsCRP was low (<2 mg/L) and there was no specific [^11^C]PK11195 uptake in the carotids (Fig. 4D).

In the brain of HC animals, [^18^F]FDG and [^11^C]PK11195 uptake showed different patterns of localized hyperfixation (Fig. 4E−F). In contrast to previous observations in aged atherosclerosis mouse models,^23^^,^^24^ no gadolinium enhancement was observed in these regions or in periventricular zones (Fig. 4G). Compared with young NHPs (Fig. 4H), [^11^C]PK11195 showed pronounced focal uptake zones, especially in the frontal cortex. In animals with active carotid lesions similar to clinical cases, TSPO-positive neuroinflammation in the frontal cortex was more focally distributed when compared with high glucose metabolism. Interestingly, two NHPs presented signs of brain degeneration on anatomical MRI, such as ventricle enlargement and hippocampus atrophy (Fig. 4I−J). In high resolution MRI, the carotid vessel wall area of HC NHPs was two times thicker compared with SD animals and presented signs of inflammation on post-gadolinium enhancement (Supplementary Fig. 5 A and B). An example of a vulnerable carotid plaque (MRI and pathology) with cerebrovascular MRI signs is presented in Supplementary Fig. 5C−M and the overall neurovascular MRI assessment in Supplementary Table 6.

**Figure 5:**
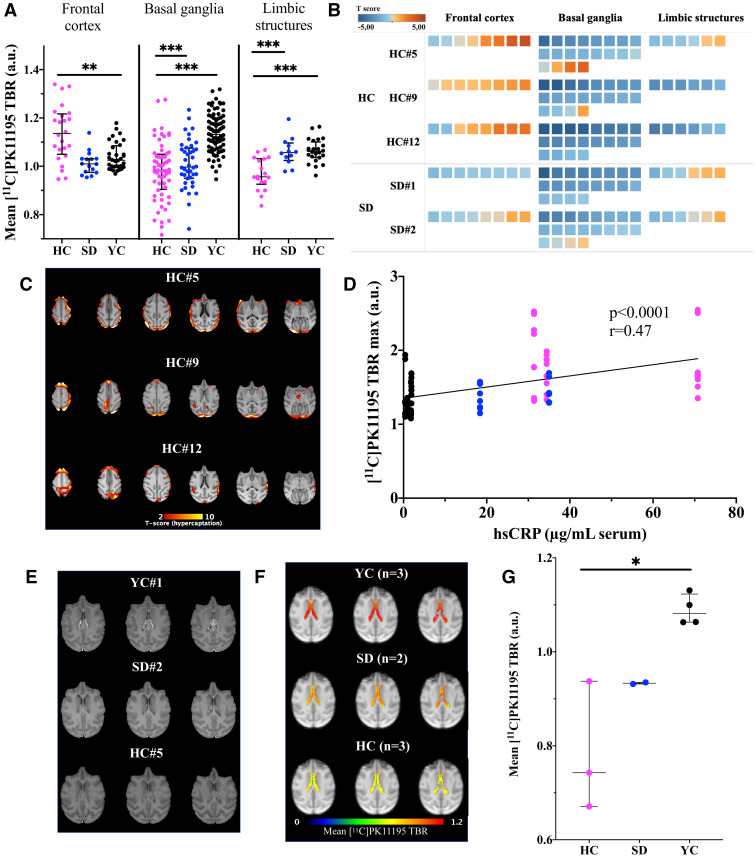
**PET [^11^C]PK11195 analysis shows cortical brain regions with hyperfixation in HC animals with correlation with hsCRP and impaired CP activity.** [^11^C]PK11195 fixation in several brain regions shows significant differences between animal groups (**A**—3 HC, 2 SD, 4 YC NHPs, each with 8 frontal cortex, 20 basal ganglia, 6 limbic structures regions, Kruskal−Wallis ANOVA with Dunn’s multiple comparisons **P = 0.004; ***P < 0.0001). *T*-score heatmap of [^11^C]PK11195 fixation sampled in selected brain regions revealed hyper and hypocaptation of [^11^C]PK11195 (TSPO expression) (**B**). *T*-score maps in 3 HC animals (HC#5, HC#9 and HC#12) illustrate the presence of frontal foci of inflammation (**C**), with a significant correlation between circulating hsCRP level and [^11^C]PK11195 fixation in frontal brain regions (**D**, *n* = 72 regions in 3 HC, 2 SD, 4 YC NHPs, *r* = 0.47, *P* < 0.0001). Representative examples with 3 brain slices of gadolinium contrast-enhanced MRI (**E**) and averaged [^11^C]PK11195 fixation in the 3 groups (**F**), and [^11^C]PK11195 fixation in each group in the lateral ventricles (**G**, Kruskal−Wallis ANOVA with Dunn’s multiple comparisons, *P = 0.042). CP in YCs (YC, *n* = 4) was positive on both MRI and PET, whereas decreasing activity was shown in old animals under standard diet (SD, *n* = 2) and almost complete extinction in old atherosclerotic high-cholesterol diet (HC, *n* = 3) animals. HC = high cholesterol group; hsCRP = high-sensitivity C reactive protein; NHP = non-human primate; SD = standard diet group; YC = young control group.

We further analysed regions with higher or lower [^11^C]PK11195 uptake, by developing a dedicated post-processing pipeline in three steps using the YC dataset and the cynomolgus atlas^17^ (Supplementary Fig. 1). Higher uptake was confirmed in the frontal cortex in HC animals (Fig. 5A−C; Supplementary Fig. 6A−D). [^11^C]PK11195 level in frontal brain regions correlated significantly with the systemic hsCRP level (Fig. 5D, *r* = 0.47, *P* < 0.0001). Neuroinflammation was further confirmed by gene analysis from the frontal lobe, with an association between TSPO, Ccl2/Mcp-1 and cd3e mRNA levels (Supplementary Fig. 4). Interestingly, there was also an association between Il-6 and Hk1, a plausible inflammatory contribution to the observed high [^18^F]FDG uptake in the frontal cortex.

Of note, localized areas of [^11^C]PK11195 hypofixation were also found (Supplementary Fig. 6E) (caudate, putamen, striatum), consistent with MRI brain abnormalities and with previous clinical observations in chronic neuro-inflammatory diseases.^25^ In aged animals under standard diet, foci of hyper- and hypo-fixation were also present (Supplementary Fig. 6 C−E), although less pronounced, which may be linked with cerebrovascular aging. CP, the gateway for neuroinflammation resolution,^26^ exhibited decreased gadolinium contrast-enhancement on MRI in aged SD animals compared with YCs, and no contrast-enhancement in HC animals (Fig. 5E). Moreover, CP activity was also markedly lower in aged versus young animals and was even cold on [^11^C]PK11195 PET imaging in HC animals (Fig. 5F−G).

## Discussion

We developed a model of active atherosclerosis in aged NHPs with systemic and vascular inflammation, metabolic syndrome with cardiovascular events and neuroinflammatory consequences. The diet-induced atherosclerosis model (high fat and 0.5% cholesterol diet for 2 years) has been described in earlier studies in cynomolgus monkeys.^27^ This model reproduced many features of human atherosclerosis, including sudden death, severe dyslipidaemia, advanced carotid and coronary plaques with downstream events. Using PET imaging, we further showed systemic and vascular inflammation, haematopoietic organ activity, and fat inflammation. These imaging features were recently demonstrated in patients.^20^ Vascular inflammation was characterized by the same imaging and gene expression signature as in human carotids, with high-risk cases. Finally, neuroinflammation in the frontal cortex, brain atrophy and impaired CP activity were demonstrated for the first time in an atherosclerosis model with gyrencephalic brain. As inflammation is a pivotal process in the aging context of atherosclerosis and stroke, this new neuroimaging signature will be essential for therapeutic decision-making in the era of immunotherapy targeting inflammaging and risk of intercurrent infection.

So far, there is no accepted model of vulnerable plaque sharing the main features of the downstream consequences of the human disease. In a mouse model of advanced atherosclerosis, we previously found active neuroinflammation in CP and periventricular areas in aged ApoE^-/-^ mice under high-fat diet. It was associated with systemic inflammation, oxidative stress and premature death.^23^^,^^24^ However, there is no plaque rupture in this model inducing downstream lesions. Among large animal models, pig is well suited to the study of coronary plaque and biomechanical factors leading to plaque rupture.^28^ However, due to vessel geometry, pig models do not develop natural carotid atherosclerosis and do not lead to embolic stroke, because of their rete mirabile.^29^ The cynomolgus macaque under atherogenic diet quickly develops a dyslipidemic blood profile and multi-site atherosclerosis progression, as in humans. The coronary and carotid lesions reproduce the plaque location and morphology observed in clinical studies, as their vessel geometry is very close to that found in humans.^9^ Moreover, this model is characterized by cardiovascular mortality, non-fatal myocardial and cerebral infarction and high systemic inflammation, with elevation of blood biomarkers of systemic inflammation similar to at-risk patients.

The innate and adaptive immune system is a key player in vulnerable plaque.^1^ In contrast to mouse models,^30^ this NHP atherosclerosis model is the only one to display the same chemokine and cytokine armamentarium as in human atherosclerosis.^19^ In NHP and patient carotid samples, vascular inflammation was characterized by higher expression of genes related to metabolism, inflammatory and anti-inflammatory macrophages and lymphocytes. In NHPs, these associations were also found in two aortic locations. Moreover, they were also directly associated with TSPO and Hk1, confirming the relevance of these imaging markers in vascular inflammation. Inflammation was present in adipose tissues, a signature of the metabolic syndrome. The relationship between M1, M2 and lymphocyte gene expression was also confirmed there.

Translation issues arise in the study of innate immune cells in advanced mouse models of atherosclerosis^31–34^: monocyte/macrophage M1/M2 subtypes have different phenotypes and hsCRP, the established biomarker in both clinical and large animal pre-clinical studies, is not relevant in mice. Defective autophagy, a mechanism linking aging macrophage dysfunction and hyperlipidaemia in the aging context,^22^^,^^35^ as well as weakened antioxidant defences and mitochondrial dysfunction^36^ were recently demonstrated in atherosclerosis, suggesting potential new therapeutic targets. However, targeting immunity should not impair host defences against infection,^22^^,^^37^ especially in aged subjects, where infection is one of the most frequent complications after an acute event, and the major cause of hsCRP peak after stroke. Insufficient knowledge of the dynamic immune response in humans is a serious limitation on clinical development of new immunotherapies in cardiovascular diseases and stroke.^38–40^ This difficult context calls for more relevant preclinical models of atherosclerosis, and the NHP model is especially suited to evaluate the efficacy of new candidate drugs.^22^^,^^38^

Earlier works with cynomolgus monkeys fed an atherogenic diet did not found advanced intracerebral arteries atherosclerosis.^27^ Our study is performed in older animals and we revealed gadolinium-enhancement in extracranial carotids (Supplementary Fig. 5). Focal spots at the level of intracranial arteries remain difficult to confirm without dedicated high spatial resolution MRI. In older humans, intracerebral atherosclerosis is mainly reported in the middle cerebral artery^41^ with consequences such as impaired vaso-reactivity or microemboli detached from atherosclerotic plaques. Carotid and intracerebral arteries can be both responsible of watershed infarct or transient ischaemic attacks affecting different brain regions.^42^ Kaczmarz et al.^42^ using perfusion MRI described the higher variability of watershed infarct localization in patients with carotid stenosis. Therefore, it is a challenging task to obtain individual mapping of cerebral vascular territories even with dedicated perfusion imaging. Microemboli generated by atherosclerotic plaque erosion may contribute to intracerebral endothelial activation, inflammation and activation of the complement pathways.^43^ Ischaemic events downstream to these atherosclerotic lesions likely correspond to repeated ischaemia and reperfusion after spontaneous thrombolysis. As such, they can induce transient blood**−**brain barrier permeability and focal microglia activation. In stroke, blood**−**brain permeability leakage is an early and transient event after ischaemia and reperfusion^44^^,^^45^ whereas TSPO activation is increased 1−3 weeks later.^46^^,^^47^ In the cynomolgus monkey, the frontal cortex is first in line for ischaemia after middle cerebral artery occlusion^48^ which could explain TSPO activation in this region. Our finding of a positive relationship between frontal cortex TSPO activation and peripheral hsCRP level indirectly confirm its atherosclerotic origin. In a small clinical study, Thackeray et al. also reported frontal cortical PK11195 hyperfixation after an acute coronary plaque rupture with myocardial infarction. Therefore, TSPO hyperactivity in this specific region may be particularly relevant to evaluate high-risk atherosclerosis.

A recent human PET study demonstrated that active atherosclerosis was linked to systemic inflammation and enhanced regional brain glucose metabolism.^20^ In humans, FDG PET showed hypermetabolism in the amygdala and other regions under stress and various pathologic conditions. In the same context of aged animals under HC diet, we confirmed enhanced glucose metabolism in vascular atherosclerosis locations (carotid, aorta), and in brain parenchyma in the frontal cortex. Neuroinflammation was also present in this area, with no evidence of BBB damage at the time of imaging.

Similarly to a recent human study where systemic inflammation was linked to brain aging,^49^ metabolic inflammation (high hsCRP and TG associated with hsCRP) was associated with higher neuroinflammation (TSPO imaging). Interestingly, gene expression identified distinct features in the myocardium and in the brain, consistent with different mechanisms, heart remodelling and post-stroke remodelling.^3^ The role of the 18 kDa mitochondrial translocator protein (TSPO) in healthy and diseased human brain tissue is still a matter of debate; it is elevated in many cells, including microglia/macrophages, astrocytes, endothelial cells, vascular smooth muscle cells and CP epithelial cells.^50^ Immunohistochemistry showed a pattern of increased TSPO in vascular and perivascular cells in the frontal cortex of Alzheimer patients, and a vascular origin to this activation was suspected.^50^ In three patients investigated after myocardial infarction,^3^ the frontal cortex was also positive on TSPO PET, similarly to the present finding in NHPs showing myocardial fibrosis. Thus, our finding of higher TSPO/Ccl2/Cd3e co-expression gives a hint of its origin in cerebrovascular inflammation. It is now established that TSPO increases metabolic activity in microglia.^51^ This can explain the present dual positive glucose and TSPO imaging in the frontal cortex. However, the location in vascular and perivascular spaces and the phenotype and origin (microglia or recruited macrophages) of the activated cells need to be further explored.

We also characterized, for the first time, the CP on imaging in the context of aging atherosclerosis. Despite high levels of systemic inflammation and frontal neuro-inflammation, CP activity was almost absent. In atherosclerosis mouse models, CP exhibits lipid deposit and abnormal permeability, and the complement pathway was involved at this essential immunological blood**−**brain interface.^52^ In a rat model of transient ischaemic attack, CP activity on PK11195 PET images was also reported as a sign of microglial activation.^53^ Our finding of hypoactivity in some regions and the impaired CP activity may appear controversial. Yet, the complex relationship between peripheral and central inflammation using TSPO PET imaging was recently addressed in a paper from Turkheimer et al.^54^ where hypoactivity in the CP was inversely correlated to the hsCRP level. There was also a decrease of CP permeability. It was concluded that this non-homeostatic behaviour could result in secondary microglia activation in brain parenchyma and poor outcome. We indeed also observed impaired CP TSPO activity together with decrease gadolinium enhancement which could sign the chronic systemic inflammation in these aged animals with diet-induced atherosclerosis. Dysfunctional CP with defective clearance function has been suggested to be an important mechanism of poor prognosis in the aging brain.^55–58^ CP recruitment of reparative leukocytes was found to be impaired through a mechanism involving nitric oxide.^59^ This deserves future exploration using molecular PET/MRI in clinical high-risk atherosclerosis and cerebrovascular diseases.

## Limitations

The present study had some limitations, such as the small number of animals and the absence of longitudinal neuroinflammation imaging together with dedicated post-mortem brain immunohistochemistry. Recent reviews of neuroinflammation and thromboinflammation in the context of stroke^40^^,^^60^^,^^61^ confirmed the urgent need for translational and clinical investigation with neuroinflammation imaging to better understand the stage-dependent contribution of innate and adaptative immune mechanisms, so as to develop new personalized immunotherapy.

## Conclusion

We conclude that neuroinflammation is an important factor in the complex clinical picture of high-risk atherosclerosis in aging individuals, and that it can readily be reproduced in NHPs. The frontal cortex is the principal location of neuroinflammation in the gyrencephalic brain, unlike in rodent models. Also, neuroinflammation was not associated with BBB leakage at the time of observation, another distinct feature together with impaired CP activity.

In the future, systemic and neuroinflammation changes can be used as clinical imaging biomarkers of high cerebrovascular risk for personalized treatment targeting inflammation. These findings also further incite clinical studies with whole-body PET/MRI investigation of inflammation in atherosclerosis.

## Supplementary material

Supplementary material is available at *Brain Communications* online.

## Supplementary Material

fcab064_Supplementary_Data

## Abbreviations

BBB =: blood-brain barrier
CP =: choroid plexus
FDG =: fluorodeoxyglucose
FLAIR =: fluid attenuated inversion recovery
HC =: high-cholesterol diet
HDL =: high density lipoprotein cholesterol
LDL =: low density lipoprotein cholesterol
MPR =: multiplanar reconstruction
NHP =: non-human primate
SD =: standard diet
SUV =: standardized uptake value
TBR =: target to background ratio
TSPO =: translocator protein
YC =: young control
