# Supplementary material for: Cortical inflammation and brain signs of high-risk atherosclerosis in a non-human primate model
Source: Brain Commun. 2021 Apr 1;3(2):fcab064. doi: 10.1093/braincomms/fcab064 (PMC8063585; doi:10.1093/braincomms/fcab064)
Supplement: fcab064_Supplementary_Data [file fcab064_supplementary_data.pdf]

Supplementary Methods:

Pathology: immunostaining of carotid samples

Immunostaining was performed after fixation (PFA 4%) and permeabilization (PBS-5%-FBS-0.1% Triton X-100) of carotid slides. Slides were incubated 1 hour at room temperature in PBS-5% FBS to limit unspecific binding of primary antibodies. Primary antibodies (described below) were added overnight at 4°C.Then, after washing with PBS-5% FBS, secondary antibodies were applied for 2 hours at room temperature. After washing, samples were prepared to be analyzed by confocal microscopy.

We used the following primary antibodies at the indicated final concentration: mouse monoclonal IgG1 anti CD68 (Abcam ab201340, 1µg/mL), rabbit polyclonal IgG anti mannose receptor (CD206) (Abcam ab64693, 1µg/mL), rabbit polyclonal IgG anti collagen IV (Abcam ab6586, 20µg/mL), rabbit polyclonal IgG anti CD163 (Abcam ab87099, 5µg/mL).

Brain MRI analysis.

Cerebral parenchyma and vasculature were analyzed from MRI (T1, T2 FLAIR, T2*, post-contrast T1) by an experienced neurologist and graded similar to the search of border-zone or watershed infarct for human carotid atherosclerosis. Due to the high variability of cerebral vasculature in both

species, the investigation concerned larger areas and graded according to main cerebral arteries territories (subcortical areas or deep regions).

Supplementary Results:

In HC animals with dual tracer injection, $[^{11}C]$PK11195 carotid imaging correlated positively with $[^{18}F]$FDG findings (y=0.65x+0.15, r=0.89; p=<0.001) in HC animals, and negatively in SD animals (y=0.29x+2.26, r=-0.78, p=0.036), and there was a trend between Gd and $[^{11}C]$PK11195 in HC animals (p=0.07). Left/right carotid uptake difference was similar between MR and PET inflammation markers in HC but not in SD animals.

Tissue analysis of 3 arterial walls (carotid, aortic arch and abdominal aorta) of inflammatory proteins and oxidative stress markers (IL-1β, TNFα, IL6, MDA) confirmed the high-risk status of the NHPs, as they also had higher values for 2 or more inflammatory/oxidative markers out of the 4 selected. The 2 aortic locations (arch and abdomen) confirmed the association of inflammatory/anti-inflammatory gene expression (Figure S2). Of note, the 2 SD animals presented relatively elevated hsCRP levels (18 and 35 μg/ml respectively) and their corresponding inflammation imaging was also above normal values for gadolinium signal intensity enhancement, $[^{18}F]$FDG SUV and $[^{11}C]$PK11195 TBR. On histology, diffuse myocardial fibrosis was discovered in 1 SD animal (SD#2), confirming the *in vivo* inflammation findings and heatmap gene expression (Supplementary Fig. 3). Vulnerable carotid plaque phenotype was characterized by in-vivo MRI and pathological analysis, with corresponding brain signs on MRI (Supplementary Fig. 5). Neurovascular signs were found in 3 animals of the HC group (Supplementary Table 6).

Supplementary Table 1. **Cardiovascular risk factors and biological characteristics of symptomatic and asymptomatic patients**

	**Symptomatic (n=9)**	**Asymptomatic (n=10)**
**Men/Women**	8/1	9/1
**Age (years)**	70±9	68±8
**Hypertension**	6 (66%)	6 (60%)
**Dyslipidemia**	4/9	5/10
**Diabetes**	2/9	3 /10
**Smoking (past or active)**	7/9	8/10
**BMI**	28±5	26±4
**Stenosis degree (%)**	53±9.7	75.8±7.4
**Pathological findings**		
Intra-plaque hemorrhage	2/9	4/9
Large lipid core (>50%)	8/9	9/9
Thin or ruptured fibrous cap	9/9	9/9
Calcification	6/9	8/9

Abbreviation: BMI: body mass index.

Supplementary Table 2. List of primers

	**Primer Sense 5' to 3'**	**Primer Antisense 5' to 3'**
**Housekeeping**		
Actine ß	GAGCACGGCATCGTCACCAA	ACAGCCTGGATGGCCACGTA
**Metabolism Imaging**		
Hk1	TCTGAATAGCACCTGCGATG	CGTCTGGTGCATGATTCTGG
TSPO	CTCCTACCTGGTCTGGAAAG	ACCAACAGGAGATCCACCAG
PPIF	GGCTACAAAGGCTCCACCTTC	GAAAGCGGCTTCCGTAGATG
**Macrophages**		
CD14	GCCGCTGTTTAGGAAAGAAG	GATGAGGTTCGGAGAAGTTG
CD68	GACACCTCAGCTTTGGATTC	TCTCGAAGGGATGCATTCTG
**Inflammatory Mφ**		
IL1ß	ATCTAGACCTCTGCCCTCTG	CAAGCGTCGTTATTGCGTGT
TLR4	TCCTGCGTGAGACCAGAAAG	GTTCTGGAAAACGGAGGAAG
CCL2	GAAGAATCACCAGCAGCAAG	GTTTGGATTTGCTTGTCCAG
IL-6	ACACATTCGGTACATCCTCG	TGTTCTGGAGGTACTCTAGG
TNFα	TGACAAGCCTGTAGCCCATG	GAGGTACAGGCCTTCTGATG
CXCL9	TCTTGGGCATCATCTTCCTG	CAAGGATTGTAGGTGGATAG
IL17RA	CCACAGTTGTTTGAGCACA	GCATTTCTGGGCAGGAAACA
IL22RA1	TCTGCTCCAGCACGTGAAAT	ACCGTCAGGTTGCAGGACTT
**Anti-inflammatory Mφ**		
IL1Ra	GGAGAAAACCCAGCAAGATG	CATCACCAGACTTGACACAG

CCR2	CTGCCTGAGAAAGCCATAAG	AGATGAACACCAGCGAGTAG
IL10	CGTGGAGCAGGTGAAGAATG	CCCTGGATCTGATTTTGGAG
Clec7a	CAGGAGCAGAAAGAACAGAG	CCCAAAATCACAGCAATGAG
IRF4	ATCTCGGACCCGTACAAAGT	AAGGCGTTGTCATGGTGTTAG
CD163	ACTCCAAAATCCAGGCAACA	GCTTCACTTCAACACGTCCA
**T cells**		
CD3ε	AGCACAATGGCAACACAATG	CTTGCCTTCAGGTAGAGATG

Supplementary Table 3: MRI sequences and acquisition parameters.

MRI Sequence	Voxel size (mm^3)	TR/TE (ms)	Bandwidth (Hz/pixel)	Flip Angle (degree)	FOV (mm)
**TOF (carotid)**	0.5x0.5x1	20/3.11	250	20	200
**T1w (carotid)**	0.32×0.32×2	260/5.8	868	180	205
**PDW (carotid)**	0.32×0.32×2	262/23	868	180	205
**TWIST (carotid)**	0.7×0.7×1.2	3.0/1.17	750	25	306
**FLAIR (brain)**	0.6×0.6×0.8	5000/346	744	120	180
**T2* (brain)**	0.7x0.7x20	1200/3.04	270	70	180
**T1 MPR (brain)**	0.6x0.6x0.8	2100/2.77	210	8	160

Abbreviations: FLAIR: Fluid Attenuation Inversion Recovery; FOV: Field Of View; MPR: Multiplanar Reconstruction; PDW: Proton Density Weighted; TE: Time of Echo; TOF: Time Of Flight; TR: Time of Repetition; TWIST: Time-resolved angiography With Interleaved Stochastic Trajectories.

Supplementary Table 4: mRNA expression (Median folds [1st-3rd quartiles]) and correlations between mRNA expression levels of selected vulnerable markers in NHP carotid artery. *genes related to imaging (*Hk1* for [^{18}F]FDG and *Tspo* for [^{11}C]PK11195), **anti-inflammatory markers.

	**Correlated with**		**r**	**p**
**Tspo*** 1.22 [0.31- 2.84]	Hk1*	0.22 [0.16- 0.30]	0.69	0.023
	Tlr4	2.20 [0.07- 10.66]	0.94	<0.001
	Ccl2	0.09 [0.02- 0.40]	0.74	0.013
	Il-6	0.08 [0.02- 0.13]	0.67	0.028
	Cxcl9	0.62 [0.32- 2.35]	0.74	0.013
	Il-1ra**	0.16 [0.03- 0.39]	0.79	0.005
	Ccr2**	0.24 [0.05- 0.47]	0.98	<0.001
	Cd14	0.05 [0.03- 0.11]	0.81	0.004
	Il-10	1.29 [0.13- 6.61]	0.81	0.004
	Clec7a**	1.02 [0.07- 4.35]	0.92	<0.001
	Cd3ε	3.25 [1.10- 5.33]	0.91	<0.001
	Cd68	0.05 [0.03- 0.40]	0.84	0.002
	Cd163**	0.07 [0.03- 0.16]	0.63	0.044
**Ccl2**	Cd14		0.85	0.001
	Ppif (CyD)	0.01 [0.005- 0.05]	0.64	0.040
	Hk1*		0.91	<0.001

Il-1β	0.27 [0.05- 0.58]	0.67	0.003
Tlr4		0.79	0.006
Il-6		0.83	0.003
Cxcl9		0.91	<0.001
*Il-1ra***		0.9	<0.001
*Ccr2***		0.88	0.007
Cd14		0.86	0.001
*Clec7a***		0.87	<0.001
Cd3ε		0.79	0.006
*Ccr2***		0.88	0.007
Cd14		0.86	0.001
*Clec7a***		0.87	<0.001
Cd3ε		0.79	0.006
Cd68		0.84	0.002
Cd163		0.88	<0.001
*Irf4***	0.1 [0.04- 0.23]	0.7	0.020

Supplementary Table 5: mRNA expression (Median folds [1st-3rd quartiles]) and correlations between mRNA expression levels of selected vulnerable markers in carotid endarterectomy of symptomatic and asymptomatic patients (n=19).* genes related to imaging (*Hk1* for [18F]FDG and *TSPO* for [11C]PK11195), ** anti-inflammatory markers

	**Correlated with**		**r**	**p**
Tspo* *2.34 [1.39- 3.94]*	*Hk1**	0.04 [0.02- 0.05]	0.84	<0.001
	Ppif (CyD)	0.03 [0.01-0.05]	0.87	<0.001
	Cd14	0.16 [0.05-0.45]	0.9	<0.001
	Cd68	0.65 [0.16- 1.15]	0.88	<0.001
	Il-1β	0.51 [0.13- 1.02]	0.78	<0.001
	Tlr4	0.07 [0.04-0.18]	0.87	<0.001
	Ccl2	0.46 [0.02- 1.33]	0.94	<0.001
	Il-6	0.00 [0.00-0.02]	0.74	<0.001
	Cxcl9	4.45 [1.65- 6.03]	0.72	<0.001
	Il-17ra	2.7 [1.35- 3.72]	0.93	<0.001
	Il-22-ra1	3.87 [2.00- 6.33]	0.69	<0.001
	*Il-1ra***	0.01 [0.00- 0.02]	0.7	<0.001
	*Ccr2***	1.44 [0.06- 2.37]	0.92	<0.001
	*Il-10***	0.76 [045- 1.91]	0.93	<0.001
	*Clec7a***	1.17 [0.49- 2.53]	0.92	<0.001
	*Irf4***	7.00 [2.22- 19.67]	0.60	<0.001

	*Cd163***	0.14 [0.05- 0.22]	0.88	<0.001
	Cd3ε	3.74 [1.46- 4.83]	0.85	<0.001

Supplementary Table 6: Neurovascular MRI analysis

	HC (n=7)	SD (n=2)
Silent brain infarct (FLAIR)	2/7[#]	0/2
Microbleeds (T2*)	1/7[##]	0/2

[#] left subcortical insular stroke (HC#9) and frontal left periventricular hypersignal (HC#12) [##] left lenticular microbleeds (HC#1)

Supplementary Figure 1: The translational multimodal image analysis pipeline was designed for vascular and brain characterization of active atherosclerosis with neuroinflammation. Post-processing pipeline for vascular (**A-E**) and brain (**F**) image analysis. For ultrasound, carotid analysis was performed at the carotid bulb by an expert cardiologist (**A**); CT and MR angiography (**B, D**) were used for registration of the carotid vessel wall between PET and MR (**C, E**); regions of interest were drawn on high-resolution MRI (**E**). PET and MRI brain images were processed in 3 steps (**F**): in Step 1, 3D T1 was used for brain extraction, segmentation and registration to the standard space and analysis in the 79 atlas regions; in Step 2, [^{18}F]FDG and [^{11}C]PK11195 tracer was quantified after PET registration to the T1 space; and in Step 3, [^{11}C]PK11195 results were compared to the available control database and T-score maps were computed using SPM.

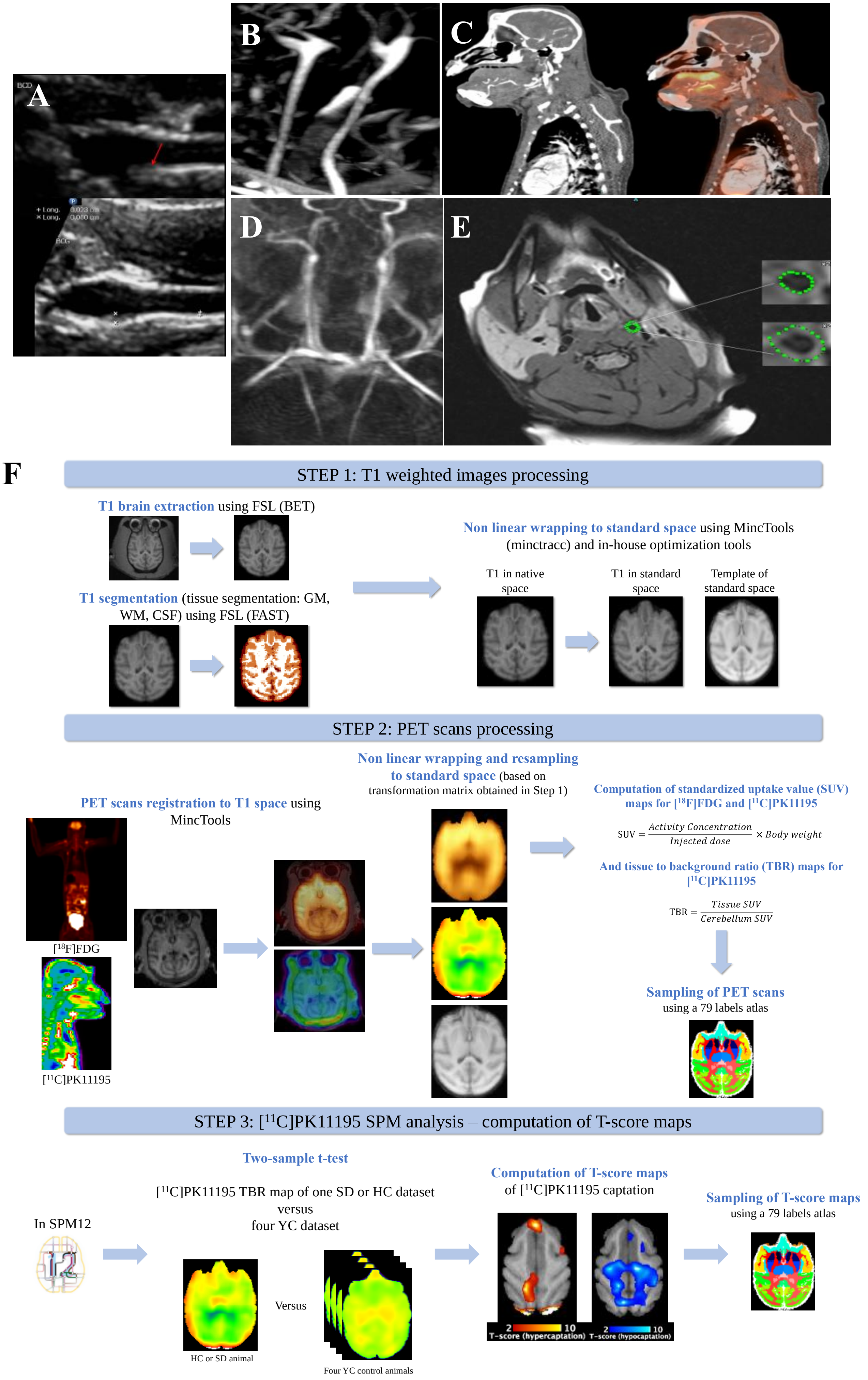
A
B
C
D
E
F
STEP 1: T1 weighted images processing
T1 brain extraction using FSL (BET)
T1 segmentation (tissue segmentation: GM, WM, CSF) using FSL (FAST)
Non linear wrapping to standard space using MincTools (minctracc) and in-house optimization tools
T1 in native space
T1 in standard space
Template of standard space
STEP 2: PET scans processing
PET scans registration to T1 space using MincTools
Non linear wrapping and resampling to standard space (based on transformation matrix obtained in Step 1)
Computation of standardized uptake value (SUV) maps for [18F]FDG and [11C]PK11195
$\text{SUV} = \frac{\text{Activity Concentration}}{\text{Injected dose}} \times \text{Body weight}$
And tissue to background ratio (TBR) maps for [11C]PK11195
$\text{TBR} = \frac{\text{Tissue SUV}}{\text{Cerebellum SUV}}$
Sampling of PET scans
using a 79 labels atlas
[18F]FDG
[11C]PK11195
STEP 3: [11C]PK11195 SPM analysis – computation of T-score maps
Two-sample t-test
[11C]PK11195 TBR map of one SD or HC dataset
versus
four YC dataset
In SPM12
Versus
HC or SD animal
Four YC control animals
Computation of T-score maps
of [11C]PK11195 captation
2 10
T-score (hypercaptation)
2 10
T-score (hypocaptation)
Sampling of T-score maps
using a 79 labels atlas

Supplementary Figure 2: Lipid profiles, ultrasound scores and hsCRP over follow-up confirmed active atherosclerosis in old subjects.

Body weight (**A**), plasma cholesterol level (**B**) and HDL/LDL ratio (**C**) in HC animals at 0, 1, 7, 12, 18 and 24 months after start of diet. Lipoprotein profile of 1 HC (**D**) and 1 control (**E**) NHP at T+24 months. Ultrasound score in the carotid arteries (**F**), plasma LDL-C level (**G**) and individual values of hsCRP including animals that died prematurely under HC and the 3 under SD (**G**). Significantly different from T0 (ANOVA with Dunnett multiple comparisons or Friedman with Dunn multiple comparisons, *p<0.05, **p<0.01, ***p<0.0005).

Abbreviations: HC and SD: high-cholesterol and standard diet; ns: non-significant; A.U.: arbitrary unit.

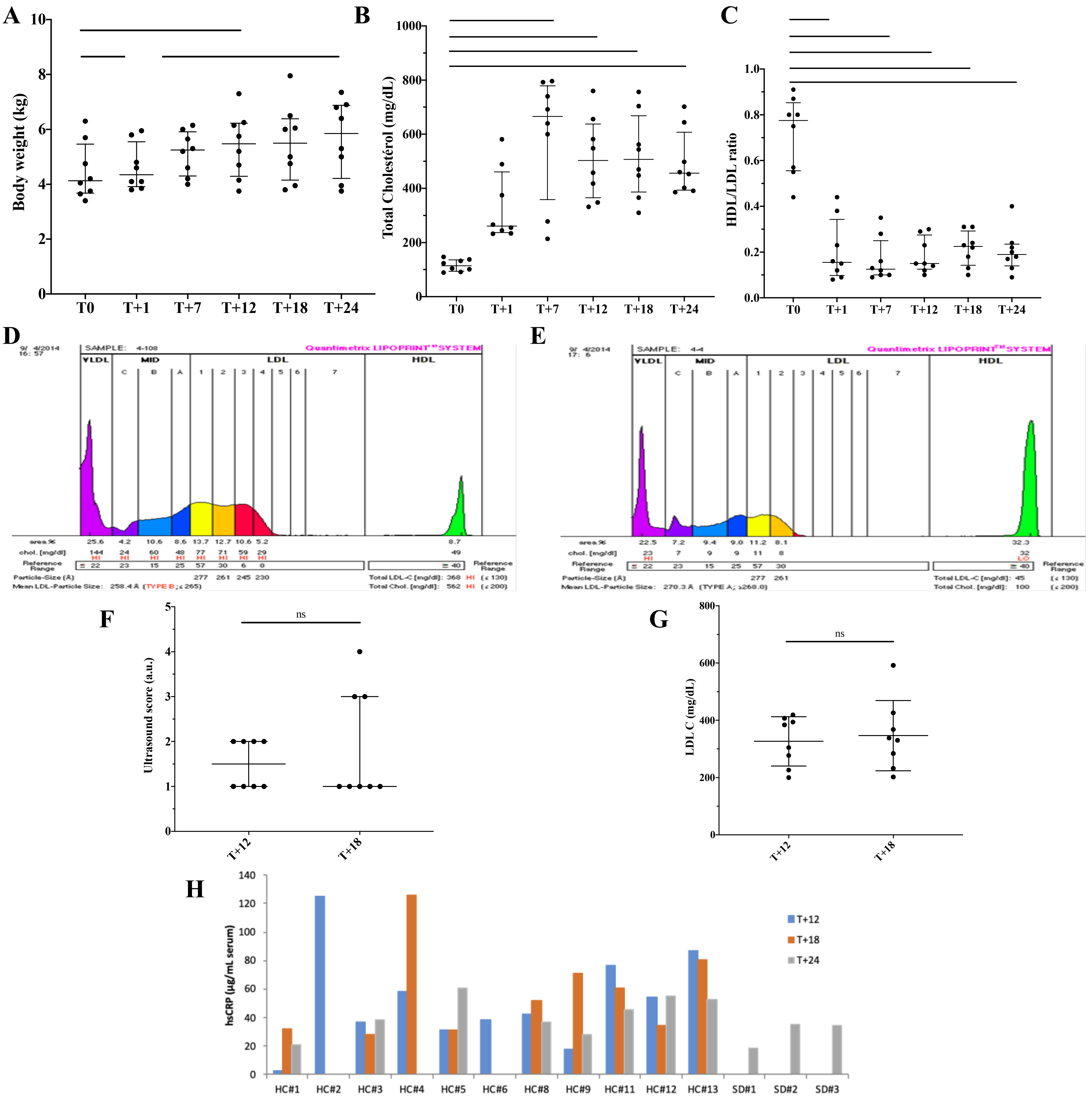
A
Body weight (kg)
T0
T+1
T+7
T+12
T+18
T+24
B
Total Cholestérol (mg/dL)
C
HDL/LDL ratio
D
E
Quantimetrix LIPOPRINT SYSTEM
VLDL
MID
LDL
HDL
F
Ultrasound score (a.u.)
ns
G
LDL C (mg/dL)
H
hsCRP (µg/mL serum)
HC#1
HC#2
HC#3
HC#4
HC#5
HC#6
HC#8
HC#9
HC#11
HC#12
HC#13
SD#1
SD#2
SD#3

Supplementary Figure 3: Carotid histology and inflammation gene expression in two other aortic locations.

Carotid histological features associated with high and low risk profiles in NHPs (**A**) and in asymptomatic patients (**B**). Masson trichrome, Hematoxylin Eosin and Oil Red O carotid staining in HC#1 (high risk, left column) and HC#3 (low risk, right column) NHP (**A**), and Masson trichrome, CD68 and Perls staining of endartectomy samples in AS#6 (high risk, left) and AS#1 (low risk, right) asymptomatic patients (**B**). RNA markers in other vascular locations (aortic arch, abdominal aorta) confirmed gene expression and association found in carotids (**C-D**) (Heat map row z-score of normalized gene expression and Ward2 hierarchical clustering). Abbreviations: NHP: non-human primate.

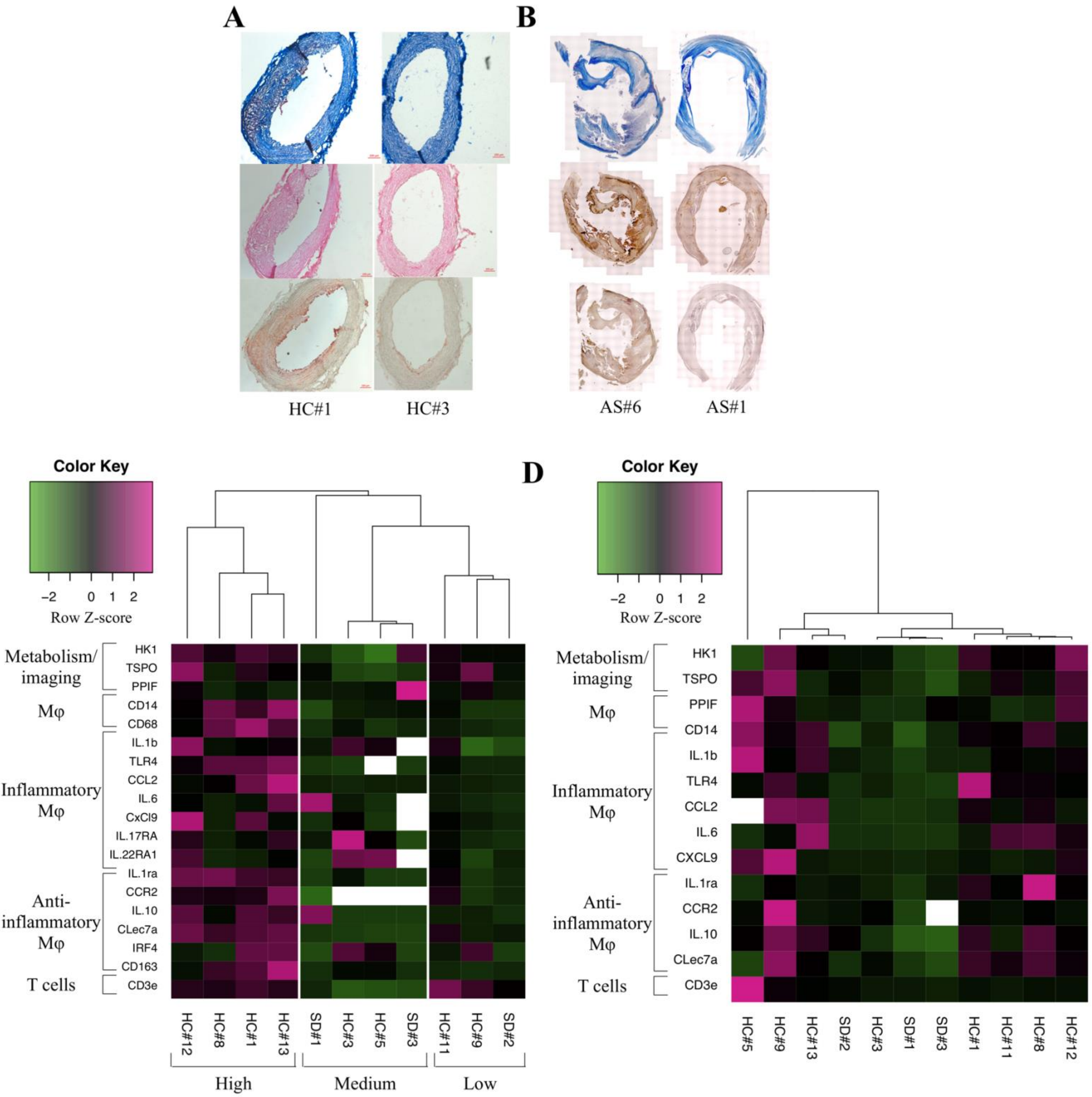
A
B
HC#1
HC#3
AS#6
AS#1
C
Color Key
−2
0
1
2
Row Z-score
Metabolism/ imaging
HK1
TSPO
PPIF
Mφ
CD14
CD68
Inflammatory Mφ
IL.1b
TLR4
CCL2
IL.6
CxCl9
IL.17RA
IL.22RA1
Anti-inflammatory Mφ
IL.1ra
CCR2
IL.10
CLec7a
IRF4
CD163
T cells
CD3e
HC#12
HC#8
HC#1
HC#13
SD#1
HC#3
HC#5
SD#3
HC#11
HC#9
SD#2
High
Medium
Low
D
Color Key
−2
0
1
2
Row Z-score
Metabolism/ imaging
HK1
TSPO
Mφ
PPIF
CD14
Inflammatory Mφ
IL.1b
TLR4
CCL2
IL.6
CXCL9
Anti-inflammatory Mφ
IL.1ra
CCR2
IL.10
CLec7a
T cells
CD3e
HC#5
HC#9
HC#13
SD#2
HC#3
SD#1
SD#3
HC#1
HC#11
HC#8
HC#12

Supplementary Figure 4: Gene expression in downstream organs (frontal cortex and myocardial apex) and in fat deposits, relationship between circulating triglycerides and hsCRP and metabolic imaging in fat deposits confirmed inflammation and metabolic syndrome.

Heatmap gene expression in brain (**A**) and in the heart (**B**) confirmed gene association and frontal cortex and myocardial inflammation in two to three subjects (**B**). Similar gene association with inflammation was also present in pericardial adipose tissue (AT) (**C**) (Heat map row z-score of normalized gene expression and Ward2 hierarchical clustering). The positive relationship between circulating triglycerides and hsCRP at the end of the study (**D**) confirmed the metabolic syndrome (r^2=0.66, $p<0.005$). It was further assessed by higher FDG uptake in visceral adipose tissue (VAT) compared to sub-cutaneous adipose tissue (SAT) (**E,F**) (F for individual results) (Kruskall Wallis with Dunn multiple comparisons, *$p<0.05$, **$p<0.01$).

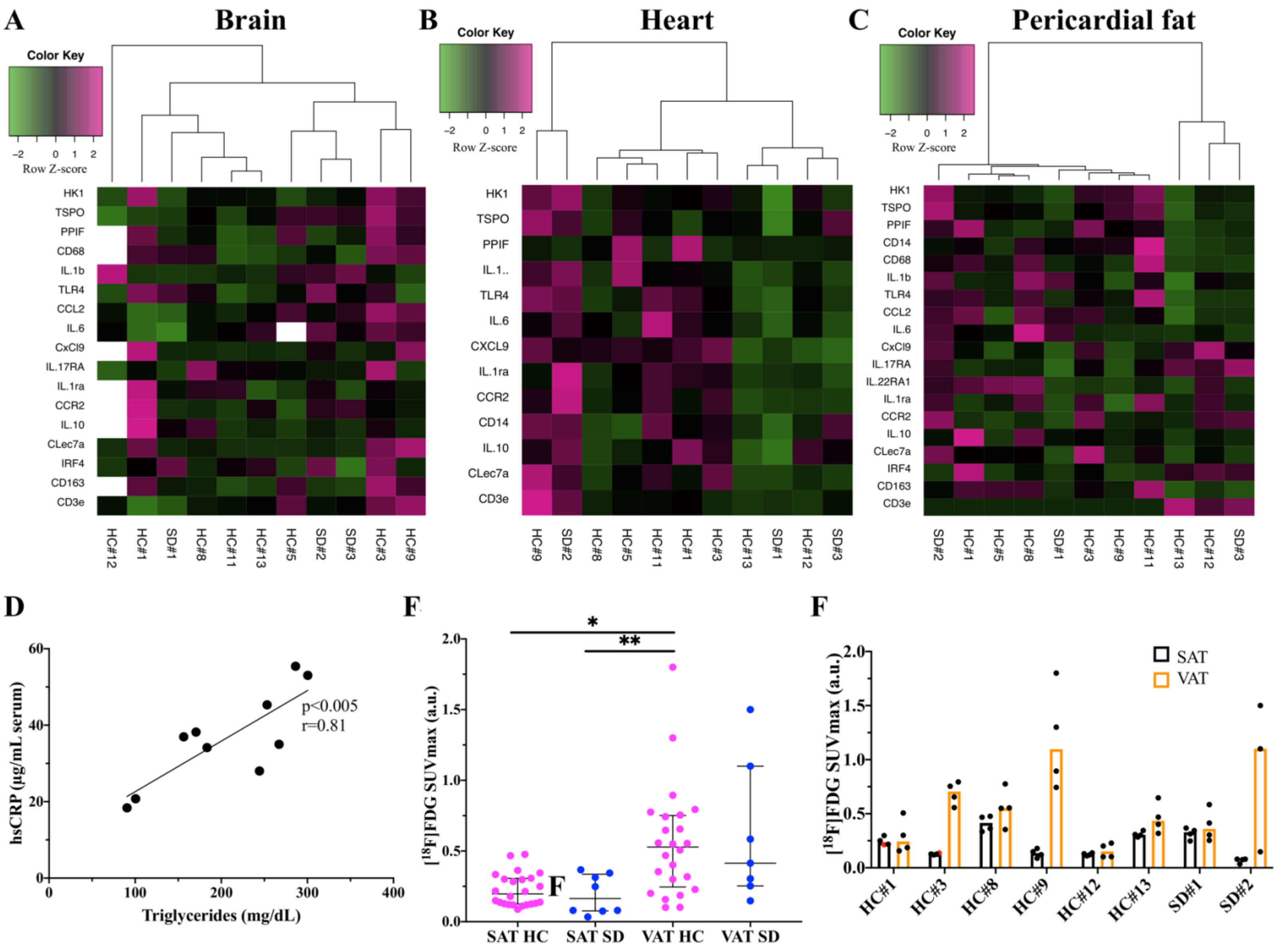

A
Brain
Color Key
Row Z-score
HK1
TSPO
PPIF
CD68
IL.1b
TLR4
CCL2
IL.6
CxCl9
IL.17RA
IL.1ra
CCR2
IL.10
CLec7a
IRF4
CD163
CD3e
HC#12 HC#1 SD#1 HC#8 HC#11 HC#13 HC#5 SD#2 SD#3 HC#3 HC#9
B
Heart
Color Key
Row Z-score
HK1
TSPO
PPIF
IL.1..
TLR4
IL.6
CXCL9
IL.1ra
CCR2
CD14
IL.10
CLec7a
CD3e
HC#9 SD#2 HC#8 HC#5 HC#11 HC#1 HC#3 HC#13 SD#1 HC#12 SD#3
C
Pericardial fat
Color Key
Row Z-score
HK1
TSPO
PPIF
CD14
CD68
IL.1b
TLR4
CCL2
IL.6
CxCl9
IL.17RA
IL.22RA1
IL.1ra
CCR2
IL.10
CLec7a
IRF4
CD163
CD3e
SD#2 HC#1 HC#5 HC#8 SD#1 HC#3 HC#9 HC#11 HC#13 HC#12 SD#3
D
hsCRP (µg/mL serum)
Triglycerides (mg/dL)
p<0.005
r=0.81
E
[18F]FDG SUVmax (a.u.)
SAT HC
SAT SD
VAT HC
VAT SD
F
[18F]FDG SUVmax (a.u.)
SAT
VAT
HC#1 HC#3 HC#8 HC#9 HC#12 HC#13 SD#1 SD#2

Supplementary Figure 5: In-vivo high-resolution carotid MRI and histology showing the carotid atherosclerotic vulnerable phenotype and the corresponding brain MRI.

MRI carotid plaque areas in HC animal were two times higher than SD animals (0.11± 0.02 versus 0.04±0.02 cm^2, 7 HC and 2 SD, 6 carotid regions per NHP) (**A**). Inflammation was evaluated in HC NHPs as signal enhancement compared to muscle on post-gadolinium MRI (1.22±0.17 a.u., arbitrary unit, 7 HC, 6 carotid regions per NHP) (**B**). SI enhancement cannot be reliably measured in SD due to small vessel wall area and partial volume effect. Example of high resolution pre and post-gadolinium carotid MRI (**C-D**) showing left carotid inflammation (high signal enhancement: 1.54) (**C,D**, arrowhead), (HC#12: No significant carotid stenosis but vulnerable left carotid plaque with irregular borders on US, MRI+ and PET+). Left carotid pathological analysis of HC#12 with Oil Red O (**E,F**) showed disrupted fibrous cap (blue box) and complex plaque with medial infiltration (green box). Immunohistochemistry confirmed disrupted cap (**G**: blue box, higher magnification, collagen IV) and complex inflammation infiltration illustrated here by staining of macrophages markers (CD163, CD68 and CD206) (**H, I, J** respectively: green box, higher magnification). Brain MRI TOF angiography of HC#12 displayed signs of intracranial carotid stenosis (**K**), pre-gadolinium T1 MRI showed hippocampus atrophy (**L**) and intracranial vascular bright spots were present on post-Gadolinium MRI (**M**).

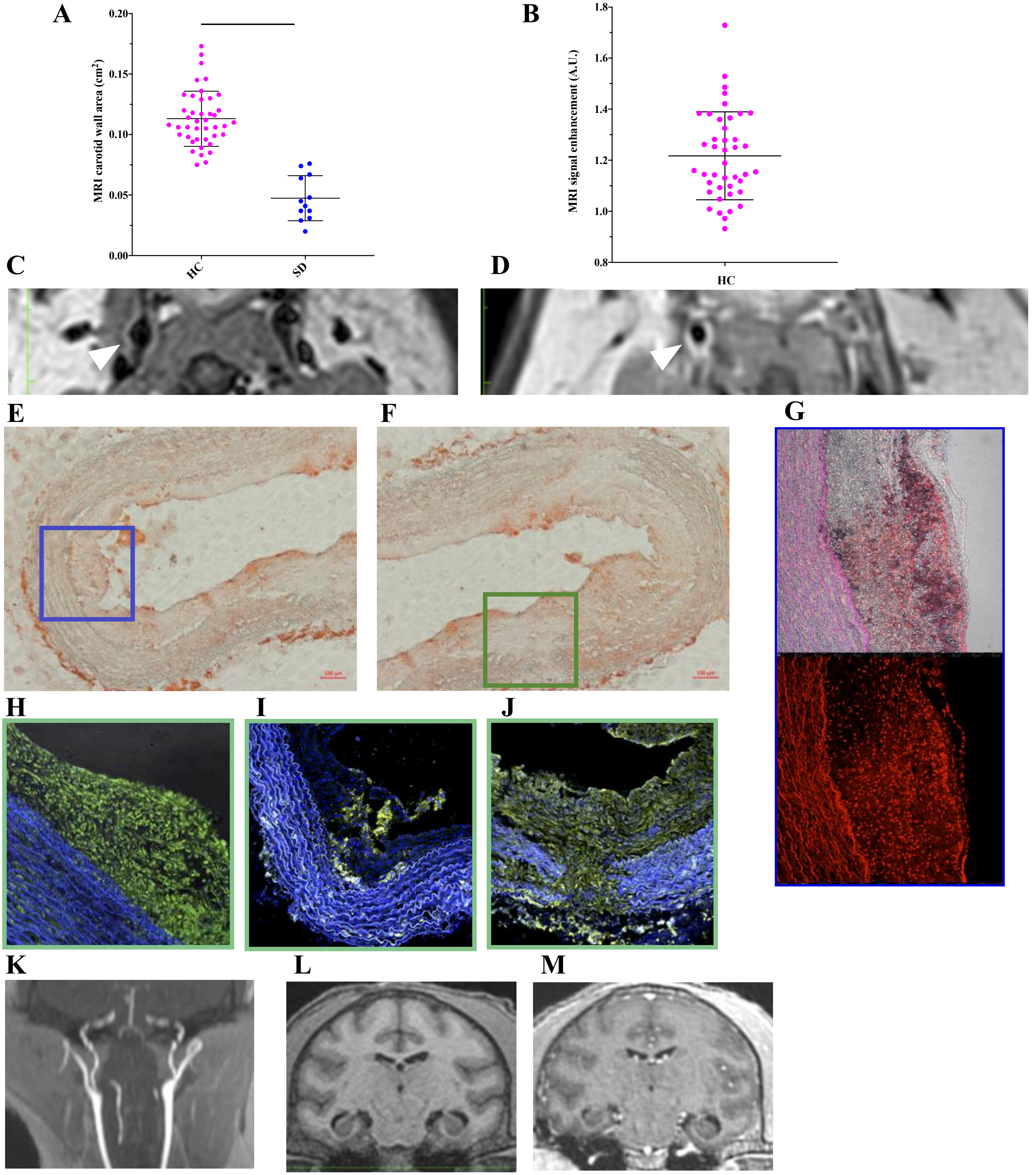
A
B
MRI carotid wall area (cm^2)
0.20
0.15
0.10
0.05
0.00
HC
SD
MRI signal enhancement (A.U.)
1.8
1.6
1.4
1.2
1.0
0.8
HC
C
D
E
F
G
H
I
J
K
L
M

Supplementary Figure 6: Active local neuroinflammation in the frontal cortex but also hypocaptation with brain atrophy are common features in old subjects with atherosclerosis. Brain PET and MRI images of the two other HC NHP (HC#5, **A**; HC#12, **B**) showing high localized FDG and PK11195 uptake in the frontal lobe (**A-B**) and brain atrophy for NHP#12 (**B**). Brain regions with PK11195 hypofixation are shown for the 3 HC animals (**C**), and hyper- and hypo-fixation for the 2 SD animals (SD#1, **D** and SD#2, **E**).

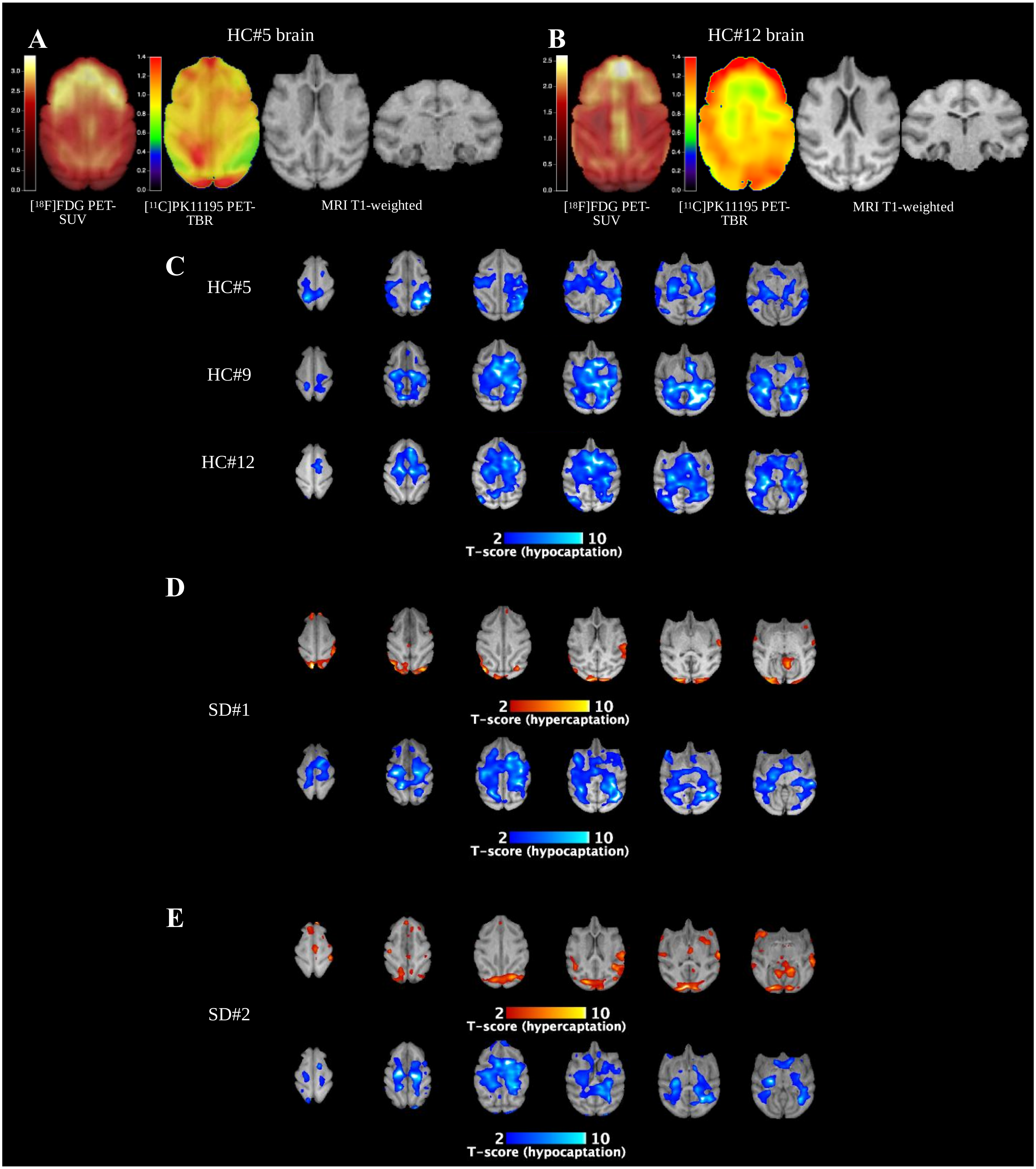
A
HC#5 brain
[18F]FDG PET-SUV
[11C]PK11195 PET-TBR
MRI T1-weighted
B
HC#12 brain
[18F]FDG PET-SUV
[11C]PK11195 PET-TBR
MRI T1-weighted
C
HC#5
HC#9
HC#12
2 10
T-score (hypocaptation)
D
SD#1
2 10
T-score (hypercaptation)
2 10
T-score (hypocaptation)
E
SD#2
2 10
T-score (hypercaptation)
2 10
T-score (hypocaptation)
